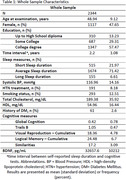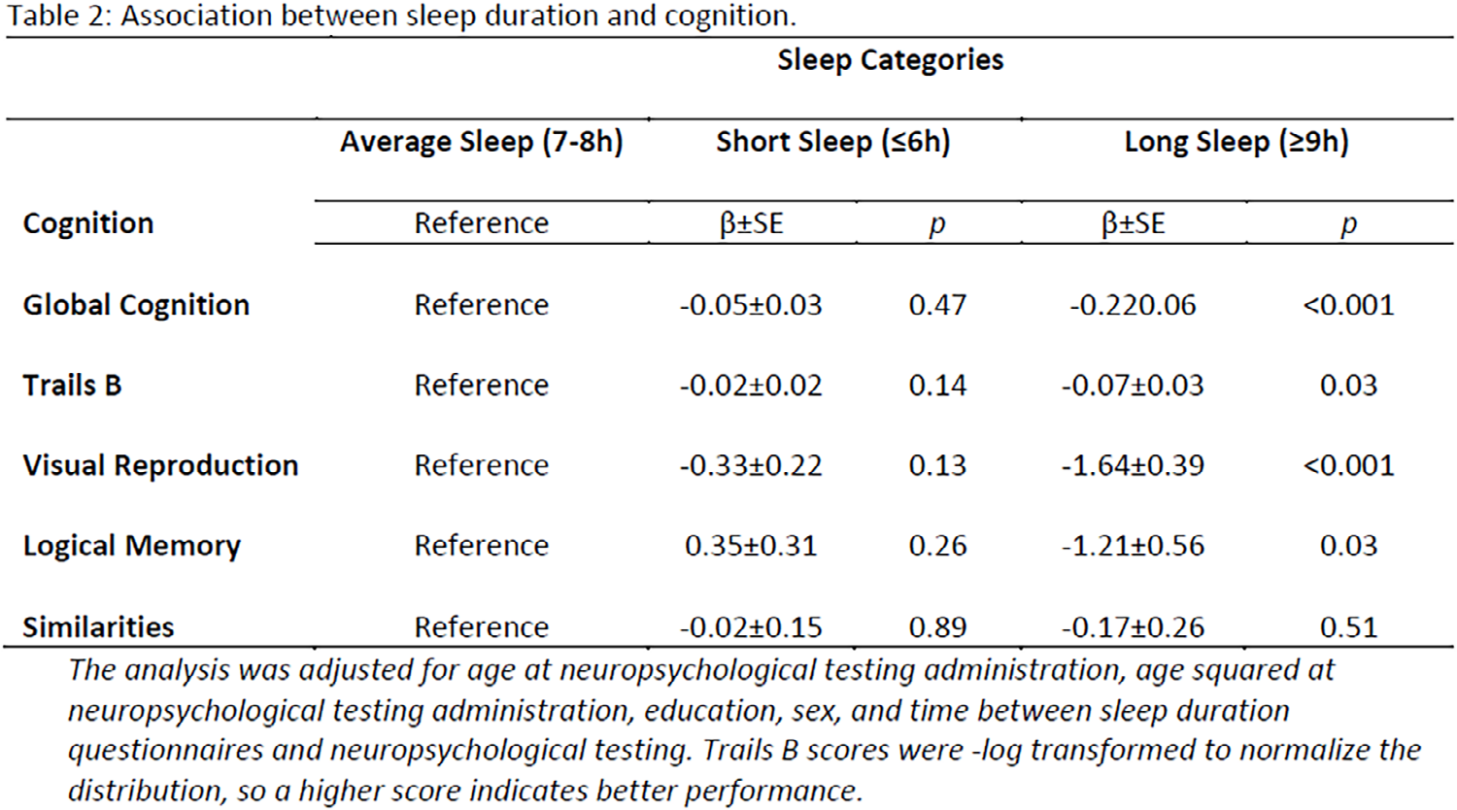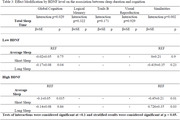# The impact of BDNF on the interplay between sleep time and cognitive function

**DOI:** 10.1002/alz.090834

**Published:** 2025-01-09

**Authors:** Joy Zeynoun, Crystal Wiedner, Andree‐Ann Baril, Vanessa M. Young, Rebecca Bernal, Alexa S Beiser, Matthew P. Pase, Sudha Seshadri, Jayandra J. Himali

**Affiliations:** ^1^ Glenn Biggs Institute for Alzheimer’s & Neurodegenerative Diseases, University of Texas Health Science Center, San Antonio, TX USA; ^2^ Research Center of the CIUSSS‐NIM, Hôpital du Sacré‐Coeur de Montréal, Montreal, QC Canada; ^3^ The Framingham Heart Study, Framingham, MA USA; ^4^ University of Montreal, Montreal, QC Canada; ^5^ Graduate School of Biomedical Sciences, San Antonio, TX USA; ^6^ School of Social and Behavioral Sciences, Arizona State University, Phoenix, AZ USA; ^7^ Glenn Biggs Institute for Alzheimer's & Neurodegenerative Diseases, University of Texas Health San Antonio, San Antonio, TX USA; ^8^ Boston University School of Public Health, Boston, MA USA; ^9^ Boston University Chobanian & Avedisian School of Medicine, Boston, MA USA; ^10^ Harvard T.H. Chan School of Public Health, Harvard University, Boston, MA USA; ^11^ Turner Institute for Brain and Mental Health & School of Psychological Sciences, Monash University, Clayton, VIC Australia; ^12^ Department of Neurology, University of Texas Health Sciences Center, San Antonio, TX USA; ^13^ Department of Population Health Sciences, UT Health San Antonio, San Antonio, TX USA

## Abstract

**Background:**

Disrupted sleep patterns have been shown to exacerbate Alzheimer's disease (AD) risk, potentially because of sleep's role in memory consolidation and synaptic plasticity. Recent evidence highlights that high brain‐derived neurotrophic factor (BDNF) levels, a protein enabling neuroplasticity and memory functions, could play a protective role in age related cognitive impairment. We examined the association between total sleep time and cognition, and BDNF levels as a potential modifier.

**Method:**

Third Generation, Omni 2, and New Offspring cohorts from the Framingham Heart Study Exam 2 (2008‐2011) were included (n=2,344; age 48(9.1) y; 48%F; Table 1). Self‐reported total sleep duration was categorized as: short sleep duration (≤6h), average sleep (7‐8h, reference), and long sleep duration (≥9h). A composite measure of global cognition was calculated from neuropsychological tests, including Trails B, visual reproduction (VR), logical memory (LM), and similarities. A multivariable linear regression estimated the association between sleep duration and global cognition, and individual cognitive tasks. We further tested effect modification by BDNF level (median split).

**Results:**

Long sleep duration was associated with worse global cognition (β±SE: ‐2.19 ±0.06; p<.001), compared to average sleep (Table 2). The association between sleep duration and individual cognitive tests are shown in Table 2. BDNF levels significantly modified the association of sleep duration with global cognition (p=0.03). Long sleep duration was associated with poorer global cognition (‐0.17±0.08; p=0.04) in persons with lower BDNF, but not in those with higher BDNF levels (Table 3). Finally, short sleep duration was associated with poorer global cognition (‐0.1±0.05; p=0.04) only among those with higher BDNF (Table 3).

**Conclusion:**

Long sleep duration was associated with poorer global cognition, an effect most notable in those with lower BDNF levels. Short sleep duration was also associated with worse global cognition, but in those with higher BDNF. An appropriate sleep duration may promote neuronal integrity and prevent age‐related cognitive disorders. Further studies shall elucidate the role of BDNF in the interplay between sleep duration and cognition.